# Formation of Platinum Catalyst on Carbon Black Using an In-Liquid Plasma Method for Fuel Cells

**DOI:** 10.3390/nano7020031

**Published:** 2017-01-31

**Authors:** Yoshiyuki Show, Yutaro Ueno

**Affiliations:** Department of Electrical and Electronic Engineering, School of Engineering, Tokai University, Hiratsuka 259-1292, Japan; show@keyaki.cc.u-tokai.ac.jp

**Keywords:** platinum (Pt) catalyst, proton exchange membrane fuel cell (PEMFC), in-liquid plasma

## Abstract

Platinum (Pt) catalyst was formed on the surface of carbon black using an in-liquid plasma method. The formed Pt catalyst showed the average particle size of 4.1 nm. This Pt catalyst was applied to a polymer electrolyte membrane fuel cell (PEMFC). The PEMFC showed an open voltage of 0.85 V and a maximum output power density of 216 mW/cm^2^.

## 1. Introduction

Polymer electrolyte membrane fuel cells (PEMFCs) use fluorinated sulfonic acid polymer (Nafion) or other similar polymers as a membrane [1]. The function of the membrane is to provide a conduction pass for protons, and to separate reactant gases. The conductivity of a proton in fluorinated sulfonic acid polymer has a high value of 0.06–0.231 S·cm^−1^ [2]. This high conductivity decreases the inner resistance of the PEMFC and increases the output power density. PEMFCs usually operate at temperature ranges of less than 100 °C which is lower than other types of fuel cells, because the polymer membrane must be hydrated to obtain proton conduction. The low operating temperature of PEMFCs allows rapid start-up and long lifetime without the use of the heat-resistant material required in other fuel cell types. Because of these advantages, the PEMFC is considered to be suited for vehicular and stationary applications [3,4,5].

The low operating temperature of PEMFCs also causes a disadvantage in that there is a slow electrochemical reaction between hydrogen and oxygen molecules. Therefore, high loading of platinum (Pt) and/or Pt-based alloy catalyst is required for both the anode and the cathode electrodes in order to enhance the electrochemical reaction and increase the output power of PEMFCs. Because of material costs related to Pt, the catalyst needs to be formed at small sizes. 1–10 nm, on a suitable carbon support in order to increase the electrochemically active surface area (EASA) of Pt and to achieve high mass activity [6,7,8,9].

The carbon support materials for Pt catalysts are widely manufactured by the pyrolysis of hydrocarbons, such as natural gas or oil fractions from petroleum processes. Valcan XC-72 is one of the most popular commercial carbon support materials for Pt catalysts in fuel cells because of its low cost and high activity [10]. Pt and/or Pt-based alloy catalysts are formed on the carbon support material by various chemical synthesis methods, such as impregnation-reduction [11], colloidal methods [12], etc. These chemical methods are suited for the mass production of Pt catalysts; however, improvements in dispersion and size distribution are still required for achieving high mass activity.

Physical syntheses, such as the spattering method, are also a promising way to fabricate small and active Pt catalysts [13,14,15]. Several authors have reported on the advantages of sputtering deposition, which are high utilization efficiency of the Pt catalyst and low Pt loading, allowing a reduction in cost. Plasma, which is generated in liquid, is also applied for forming catalysts, such as Pt and PtAu [16,17,18,19]. Their catalytic activities were also investigated using electrochemical measurements. An advantage of in-liquid plasma is operation in an open system under atmospheric pressure, while the spattering method needs a vacuum atmosphere.

In this study, Pt particles were formed on carbon black (Valcan XC-72) using the in-liquid plasma method. The formed Pt particles were applied to a fuel cell as a catalyst. The fuel cells are also characterized.

## 2. Experimental Section

### 2.1. Formation of Platinum Catalyst on Carbon Black

Carbon black (Vulcan XC-72) was used as a support material for the Pt catalyst. The carbon black was dispersed in water at a concentration of 0.0378% using a rotary mixer without the addition of a dispersion assistant. The Pt catalyst was formed on the carbon black surface using the in-liquid plasma method [19]. A glass beaker with the volume of 50 mL was used as vessel in which the in-liquid plasma was generated. A carbon black dispersion of 40 mL was added into the beaker. Two pieces of Pt wire were added as an electrode in the carbon black dispersion. The distance of the Pt electrodes was 1 mm. A unipolar pulse voltage of 2.8 kV was applied between them and the plasma was formed in the carbon black dispersion. The generated plasma spatters the surface of the Pt electrodes and disperses Pt particles in the carbon black dispersion. Therefore, Pt nanoparticles were formed on the surface of the carbon black in the dispersion. The plasma processing time was varied, up to 20 min. The carbon black dispersion was stirred using a magnetic stirrer during this process. Moreover, the carbon black dispersion was kept below a temperature of 50 °C. The formed Pt particles were characterized using a transmission electron microscope (TEM) after drying the carbon black dispersion on a micro grid. The Pt on the carbon black was heated at a temperature of 800 °C under an air atmosphere in order to burn the carbon black. The remaining Pt particles were weighed to reveal the dependence of the in-liquid plasma time on the weight of the loaded Pt on carbon black.

### 2.2. Fabrication and Characterization of Fuel Cells Using Platinum Catalyst Formed by In-Liquid Plasma

Polymer electrolyte membrane fuel cells (PEMFC) were fabricated with the Pt particles, which were formed on the carbon black surface using the in-liquid plasma method. Carbon paper with a size of 3 × 3 cm^2^ was used for the anode and cathode electrodes. The carbon black dispersion, which was applied with the in-liquid plasma, was painted on the carbon paper at a volume of 10 mL in order to load the Pt particles on the electrodes. Nafion 117 sheet was used as the polymer electrolyte for the PEMFC. A Nafion sheet and two sheets of carbon paper were sandwiched. Thermocompression bonding was carried out at a temperature of 215 °C and a pressure of 19 kg/cm^2^ for 2 min, in order to assemble the membrane electrode assembly (MEA). This process bonds the carbon papers to the Nafion sheet and enhances the conduction of protons in the MEA. Humidified hydrogen and oxygen gases were flowed into the anode and cathode electrodes of the fuel cell, respectively, at the flow rate of 1000 sccm. The fuel cell was operated at a temperature of 70 °C.

Cyclic voltammetry measurements were conducted on the fuel cell in order to estimate the electrochemically active surface area (EASA) of the Pt catalyst. The measurements involved humidified hydrogen and nitrogen gases at the anode and cathode electrodes of the fuel cell, with an applied voltage between 0 and 1.6 V at a sweep rate of 100 mV/s. The EASA of the cathode electrode was observed from the charge of hydrogen desorption from the Pt catalyst. The EASA per unit area of the cathode electrode was estimated by using the following equation [6,20]:

(1) $$\mathrm{EASA}\left({\mathrm{cm}}^{2}/{\mathrm{cm}}^{2}\right)=\frac{Q_{des}\left(\mathrm{mC}/{\mathrm{cm}}^{2}\right)}{0.21\left(\mathrm{mC}/{\mathrm{cm}}^{2}\right)}$$

where *Q_des_* the charge of hydrogen desorption on the unit area of the cathode electrode.

## 3. Results and Discussion

**Figure 1:**
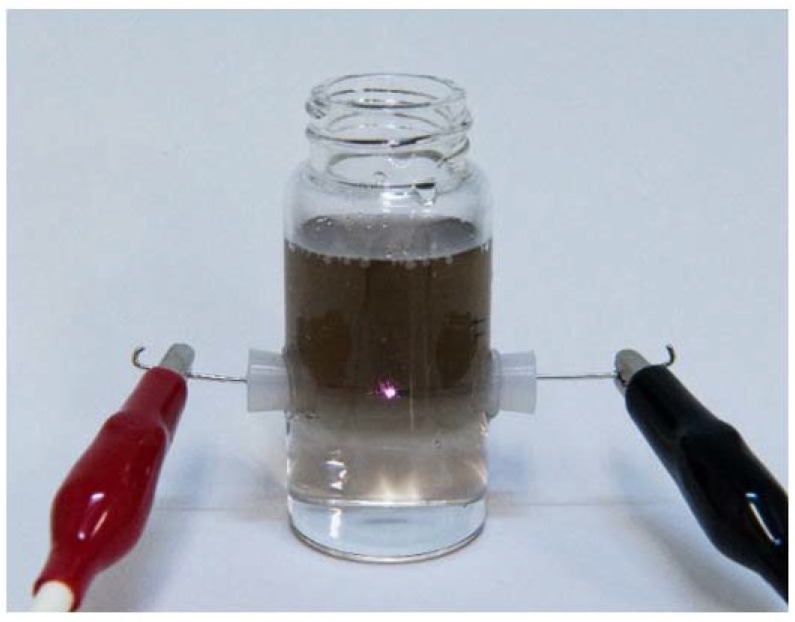
Photograph of vessel used for the in-liquid plasma process.

Figure 1 shows a photograph of the vessel used for the in-liquid plasma process. The vessel was filled with pure water instead of a carbon black dispersion when this photograph was taken. The plasma was generated between the Pt wire electrodes in water by applying a pulse voltage of 2.8 kV to the electrodes without stirring water. The upper part of the water turned black, because Pt particles were formed by the sputtering of the Pt wire electrodes. The black region became larger and darker when the process time was increased.

**Figure 2:**
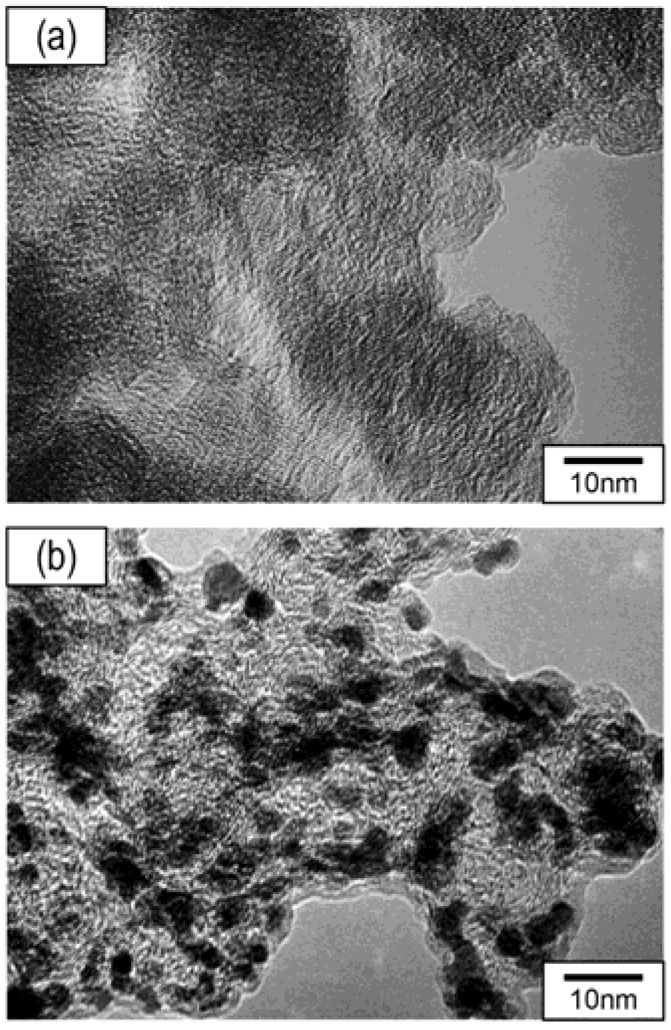
Transmission electron microscope (TEM) images of carbon black (**a**) before; and (**b**) after the in-liquid plasma process was carried out for 20 min.

Figure 2 shows TEM images of carbon black before and after the in-liquid plasma process was carried out for 20 min. The carbon black, before the in-liquid plasma process, showed aggregates of carbon particles with diameters of 10–30 nm. Small particles on the order of nanometer scale were formed on the carbon black after the in-liquid plasma process. Moreover, they were uniformly dispersed on the surface of the carbon black, with relatively narrow particle size distributions. Energy dispersive X-ray (EDX) spectroscopy showed that these particles were composed of Pt atoms.

**Figure 3:**
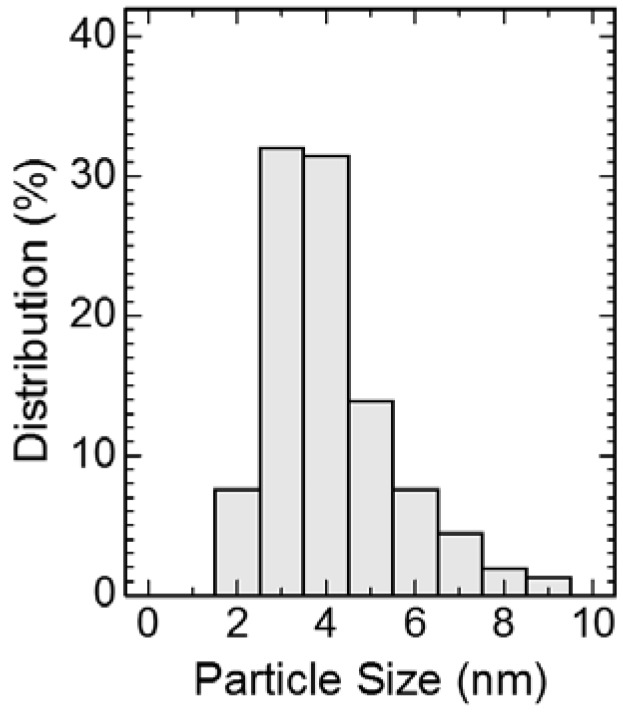
Histogram of the size distributions of the Pt particles on the carbon black.

Figure 3 shows a histogram of size distribution of the Pt particles on the carbon black. The Pt particles were 4.1 nm on average and were distributed between 2 and 9 nm. On the other hand, the Pt particles in the size range of 2–4 nm accounted for 71% of the total. This size distribution is close to the range of commercial Pt catalysts [6,21].

**Figure 4:**
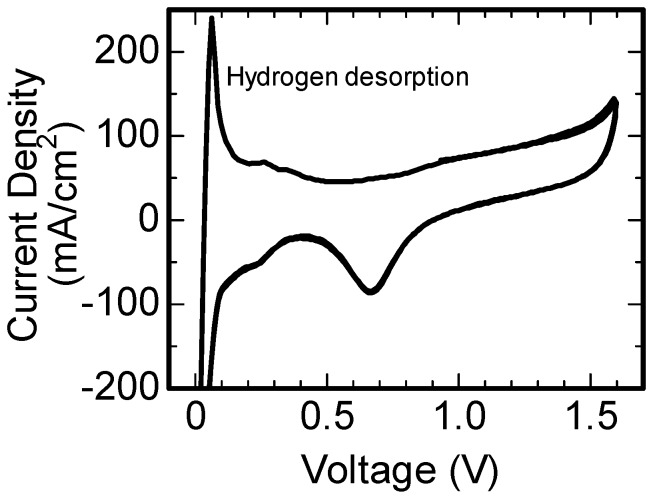
Cyclic voltammogram observed for the fuel cell, which was fabricated with the carbon black after the in-liquid plasma process was carried out for 20 min.

The fuel cell was fabricated with the carbon black after the in-liquid plasma process was carried out for 20 min. Figure 4 shows a cyclic voltammogram observed for the fuel cell. The hydrogen and nitrogen gases were flowed into the anode and cathode, respectively. The peaks related to the hydrogen adsorption and desorption on the Pt surface were observed around a voltage of 0.05 V. The electrochemically-active surface area (EASA) of the Pt was calculated from the peak of hydrogen desorption, using Equation (1). The charge of this peak for the unit area (1 × 1 cm^2^) of the MEA was 174 mC/cm^2^. Therefore, the EASA of the Pt per unit area (1 × 1 cm^2^) of the cathode electrode was estimated to be 830 cm^2^/cm^2^. The EASA per area of the MEA was available to be converted into EASA per weight of Pt particles. The converted EASA was 79 m^2^/g, which was similar to previously-reported values for the catalysts of fuel cells [6,22,23,24].

**Figure 5:**
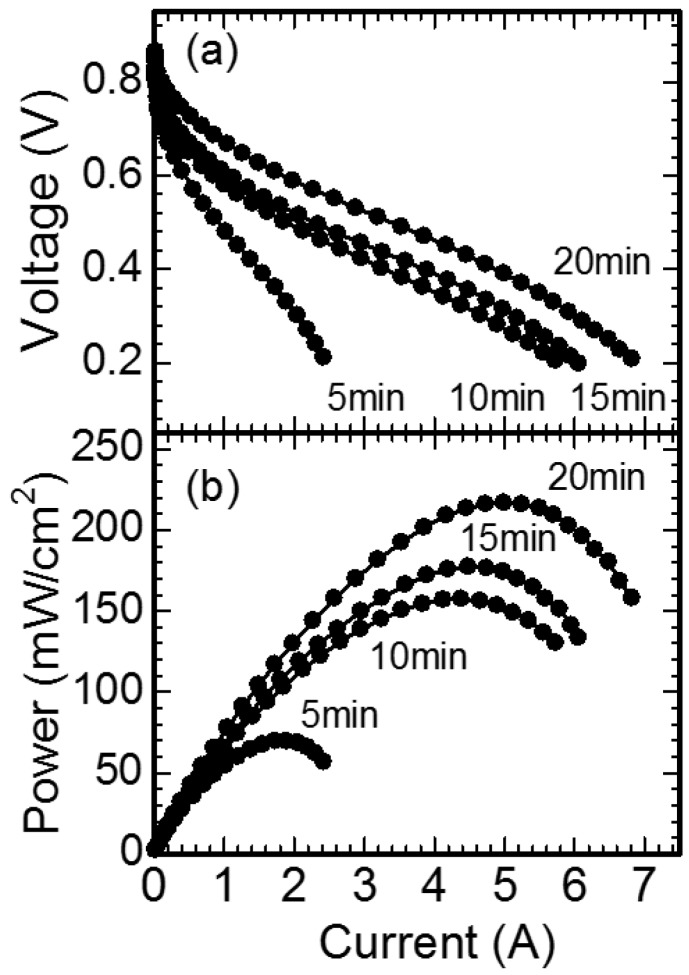
Dependence of the output current on (**a**) the output voltage; and (**b**) electric power for fuel cells fabricated with the Pt catalyst formed on carbon black using in-liquid plasma at various processing times.

Figure 5 shows the dependence of the output current on the output voltage and the electric power for the fuel cells, fabricated with the Pt catalyst formed on the carbon black by the in-liquid plasma at various processing times. The fuel cell fabricated with the carbon black, which was not applied in the in-liquid plasma, showed no output voltage, because no catalyst existed on the surface of the carbon black. The fuel cells fabricated with the Pt particles, which were formed at various processing times showed 0.85 V in open voltage. No dependence of the processing time on the open voltage was observed. This open voltage is as same as that of fuel cells formed with conventional Pt catalysts [1]. The output voltage decreased with an increase in the output current. This voltage drop mainly originated from the IR drop, which is caused by the current flowing into the internal resistance. When the processing time of the in-liquid plasma increased, the voltage of the IR drop decreased. This indicated that the internal resistance of the fuel cell decreased with an increase in the processing time of the in-liquid plasma. A maximum output power of 54 mW/cm^2^ was observed for the fuel cell with Pt catalyst formed at a processing time of 5 min. The maximum output power increased with an increase in processing time.

**Figure 6:**
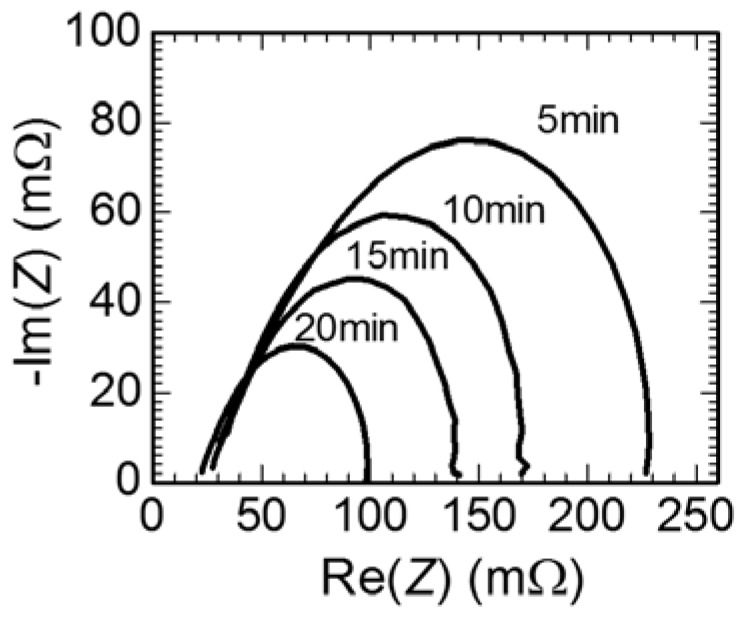
The cole–cole plots for the impedance measurements of the fuel cells fabricated using the Pt catalyst formed on the carbon black by in-liquid plasma at various processing times.

Figure 6 shows the cole–cole plots for the impedance measurements of the fuel cells fabricated using the Pt catalyst formed on carbon black by in-liquid plasma at various processing times. The fuel cells showed a semicircular curve in the negative imaginary impedance (−Im(Z)) region. The lower real impedances (Re(Z)) were approximately 30 mΩ for various processing times of the in-liquid plasma. On the other hand, the diameter of the semicircular curve decreased with an increase in processing time.

**Figure 7:**
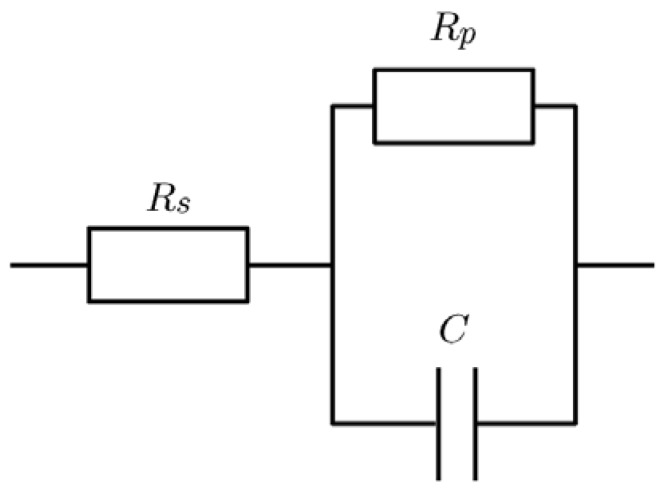
The equivalent circuit for the internal impedance of a fuel cell, which was used for estimating the series resistance (R_s_) and parallel resistance (R_p_).

The equivalent circuit for the inner impedance of the fuel cells consists of a capacitor (C), a parallel resistor (R_p_), and a series resistor (R_s_), as shown in Figure 7, because the cole–cole plots for the fuel cells were semicircular curves. The complex impedance (Z(ω)) of this equivalent circuit is calculated as a function of angular frequency (ω) using the following equation:

(2) $$Z\left(\omega \right)=R_{S}+\frac{R_{P}\left(-j\frac{1}{\omega C}\right)}{R_{P}-j\frac{1}{\omega C}}$$

The series resistance (R_s_), the capacitance (C), and the parallel resistance (R_p_) were estimated from the cole–cole plots, using Equation (2), using the least squares method.

The equivalent circuit model, as shown in Figure 7, is the Randles circuit, which is based on the reaction of a metal ion in an aqueous solution, using the process model [25,26]. Under an application of this equivalent circuit model for the characterization of a fuel cell, the series resistance (R_s_) is mainly originated from the ohmic resistance of the electrolyte; here, the proton exchange membrane. On the other hand, the parallel resistance (R_p_) originated from the charge transfer resistance, which was provided mainly by the catalytic activity of the Pt particles [25,26,27,28,29]. Moreover, in this model, the parallel resistance is the sum of charge transfer resistances in the anode and cathode electrodes, because only single parallel resistance is applied to the equivalent circuit model.

**Figure 8:**
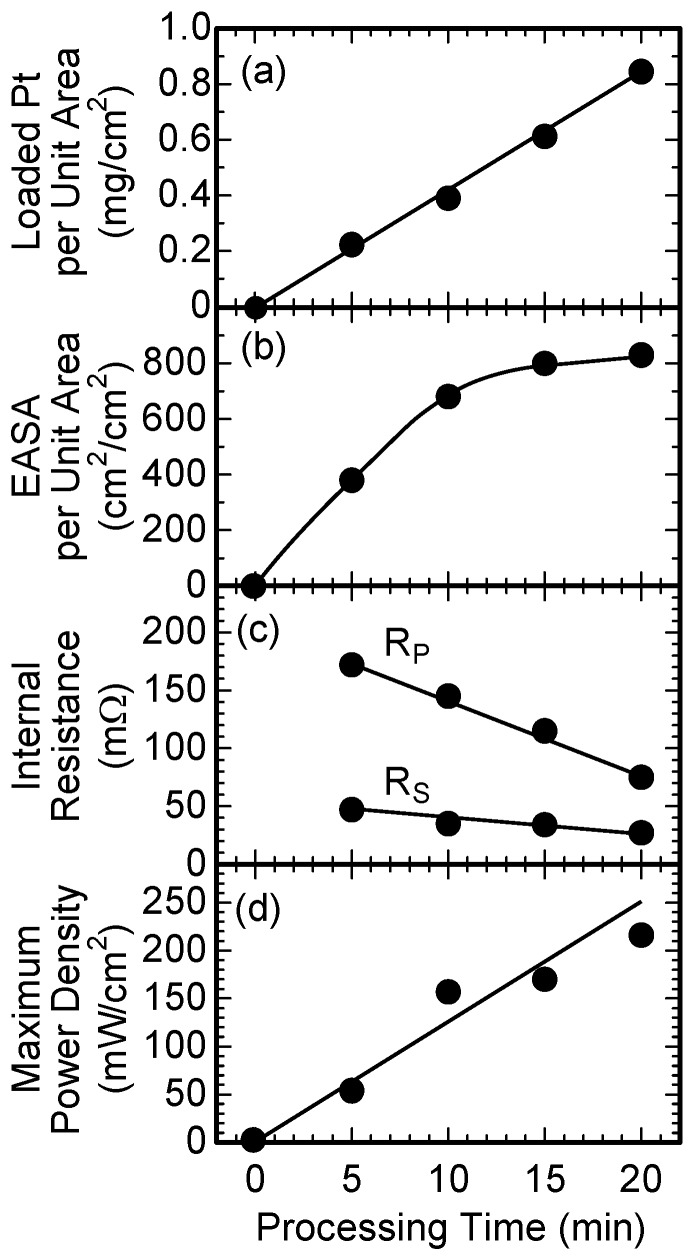
The dependence of the in-liquid plasma processing time on (**a**) the weight of the loaded Pt; (**b**) the electrochemically active surface area (EASA) of the Pt catalyst per unit area (1 × 1 cm^2^) of the cathode and electrode; (**c**) the parallel resistance (R_p_) and series resistance (R_s_); and (**d**) the maximum output power density for the fuel cells.

Figure 8 shows a summary of the experimental results. Platinum particles with the weight of 0.22 mg were loaded to the unit area (1 × 1 cm^2^) of the MEA using an in-liquid plasma processing time of 5 min. The weight of the loaded platinum linearly increased with an increase in processing time of the in-liquid plasma.

No EASA was observed for the fuel cell when the in-liquid plasma process was not carried out for active carbon, because the Pt catalyst did not exist on the surface of the carbon black. An EASA of 380 cm^2^/cm^2^ was observed for the fuel cell fabricated with carbon black, which was treated with the in-liquid plasma process for 5 min. The EASA increased to 830 cm^2^/cm^2^ with an increase in the in-liquid plasma processing time to 20 min.

The series resistance (R_s_) and the parallel resistance (R_p_) were 47 and 172 mΩ, respectively, for an in-liquid plasma processing time of 5 min. Although the processing time increased, the series resistance (R_s_) of fuel cells was not varied. On the other hand, parallel resistance (R_p_) decreased to 75 mΩ with an increase in the in-liquid plasma processing time to 20 min.

Electric power was not observed for the fuel cell assembled with the carbon black, without applying the in-liquid plasma process. On the other hand, the fuel cells with Pt particles, which were formed on carbon black by using in-liquid plasma, generated electric power. The maximum output power density linearly increased up to 216 mW/cm^2^ with an increase in the processing time.

When a unipolar pulse voltage of 2.8 kV was applied between the Pt wire electrodes in the carbon black dispersion fluid, the water of the dispersion fluid vaporized due to Joule heating and the plasma was formed. The water molecules were decomposed into hydrogen, oxygen and the radicals by plasma [17]. The surface of the Pt wire electrodes were spattered by the bombardment of energetic radicals, such as ·OH, ·H, O, O_2_^−^, HO_2_ etc. The spattered Pt atoms were condensed on the surface of carbon black and were converted into Pt particles with an average size of 4.1 nm.

Smaller particle sizes of Pt catalysts form larger surface areas, which enhance electrochemical reactions and increases the electric power generated by a fuel cell, even if the same weight of Pt catalyst is loaded. The particle size of commercial or reported Pt-related catalysts are mainly in the range of 3–15 nm [6,21]. Pt particles formed by the in-liquid plasma are the same size as the reported values. Moreover, they also have a similar value, which was reported as the optimal size for enhancing the cathodic reduction of dioxygen.

The weight of Pt sputtered by the in-liquid plasma increased with an increase in the processing time [19]. Moreover, the increase in the sputtered Pt increases the loaded Pt in MEA, and increases the electrochemically-active surface area (EASA) of the Pt particles in MEA. The increase in the EASA of the Pt particles enhances the catalytic reaction between the Pt surface and the hydrogen or oxygen molecules, which reduces the parallel resistance (R_p_) of the fuel cell. Therefore, the fuel cell, which is assembled using carbon black treated for longer in-liquid plasma processing times, shows a higher output power, because the internal resistance of the fuel cell is decreased, mainly by the reduction of parallel resistance (R_p_).

## 4. Summary

The Pt catalyst was successfully formed on the carbon black surface using the in-liquid plasma method. The particle size of the Pt catalyst was in the range of 2–4 nm. Polymer electrolyte membrane fuel cells (PEMFC) were assembled with the formed Pt catalyst. An increase in the processing time of the in-liquid plasma increased the weight of the Pt catalyst loading in the MEA of the fuel cell, and decreased the internal resistance of the fuel cell. The fuel cell, assembled with the Pt catalyst, formed by the in-liquid plasma method, generated a high electrical power density of 216 mW/cm^2^.
